# Measuring Organizational Readiness for Implementing Change in Primary Care Facilities in Rural Bushbuckridge, South Africa

**DOI:** 10.34172/ijhpm.2020.223

**Published:** 2020-11-23

**Authors:** Hannah H. Leslie, Rebecca West, Rhian Twine, Nkosinathi Masilela, Wayne T. Steward, Kathleen Kahn, Sheri A. Lippman

**Affiliations:** ^1^Department of Global Health and Population, Harvard T.H. Chan School of Public Health, Boston, MA, USA.; ^2^Division of Prevention Science, Department of Medicine, University of California, San Francisco, San Francisco, CA, USA.; ^3^Boston University School of Public Health, Boston, MA, USA.; ^4^MRC/Wits Rural Public Health and Health Transitions Research Unit (Agincourt), School of Public Health, Faculty of Health Sciences, University of the Witwatersrand, Johannesburg, South Africa.

**Keywords:** South Africa, Organizational Readiness, HIV Treatment, Implementation Science, Quality of Care

## Abstract

Meaningful gains in health outcomes require successful implementation of evidence-based interventions. Organizations such as health facilities must be ready to implement efficacious interventions, but tools to measure organizational readiness have rarely been validated outside of high-income settings. We conducted a pilot study of the organizational readiness to implement change (ORIC) measure in public primary care facilities serving Bushbuckridge Municipality in South Africa in early 2019. We administered the 10-item ORIC to 54 nurses and lay counsellors in 9 facilities to gauge readiness to implement the national Central Chronic Medicine Dispensing and Distribution (CCMDD) programme intended to declutter busy health facilities. We used exploratory factor analysis (EFA) to identify factor structure. We used Cronbach alpha and intraclass correlation (ICC) to assess reliability at the individual and facility levels. To assess validity, we drew on existing data from routine clinic monitoring and a 2018 quality assessment to test the correlation of ORIC with facility resources, value of CCMDD programme, and better programme uptake and service quality. Six items from the ORIC loaded onto a single factor with Cronbach’s alpha of 0.82 and ICC of 0.23. While facility ORIC score was not correlated with implementation of CCMDD, higher scores were correlated with facility resources, perceived value of the CCMDD program, patient satisfaction with wait time, and greater linkage to care following positive HIV testing. The study is limited by measuring ORIC after programme implementation. The findings support the relevance of ORIC, but identify a need for greater adaptation and validation of the measure.

## Background

 The excess burden of morbidity and mortality in low- and middle-income countries could be averted largely through the successful scale up of known interventions.^1^ However, proven interventions often fail to deliver health benefits when implemented at regional or national levels.^2,3^ Health system challenges, including insufficient capacity and variable leadership and management, have been identified as key barriers to implementation of evidence-based programs.^4^ Attempts to improve health services that do not address the context and implementation capacity of the health system are unlikely to succeed.^5^

 The South African health system is confronting both the ongoing epidemic of HIV and AIDS, with an estimated 7.7 million people living with HIV,^6^ and an aging population with rising incidence of non-communicable diseases that, like HIV and AIDS, require long-term care.^7^ Efficient and effective implementation of evidence-based policy is a necessity if the right to healthcare guaranteed in the constitution and the current commitment to universal health coverage are to be fulfilled,^8^ yet few tools to understand the environment for intervention uptake are available. Frameworks and tools to understand and improve intervention uptake are needed if evidence-based policy is to be translated into improved service delivery and thus better population outcomes.

 Implementation science theory provides frameworks for understanding the context of interventions within a health system.^9^ In particular, the theory of organizational readiness for change, defined as “the extent to which organizational members are psychologically and behaviourally prepared to implement organizational change,”^10^ hypothesizes that members of a group require change commitment and change efficacy – the shared resolve to change and the belief in the capacity to do so – to successfully implement a specific intervention.^11^ These are shaped by the value of the change within the organization as well as the physical and human resources available to implement the change, as illustrated in the conceptual framework in Supplementary file 1.^11,12^ Higher readiness to change should result in greater effort in implementing the change, facilitating effective implementation. Understanding readiness to change can inform preparations for implementation as well as evaluation of success and failures, although few studies have tested levels of readiness against subsequent implementation effectiveness.^13-15^

 A number of instruments to address organizational readiness have been developed and tested, with distinct sub-scales defined to suit the measure’s purpose and intended clinical setting.^14,16^ A brief measure of Organizational Readiness to Implement Change (ORIC), which contains two sub-scales that are tailored to assess commitment and efficacy related to implementation of a specific program, was developed in the United States and validated as a sensitive measure of organizational traits.^12^ It has subsequently been translated and validated for healthcare settings in Quebec, Denmark, and Switzerland.^17-19^ While there have been efforts to measure concepts underpinning organizational readiness in South Africa^20^ and there is interest in adapting and validating locally appropriate measures in both healthcare^21^ and educational settings,^22^ to date there is limited evidence of the feasibility and relevance of the brief ORIC measure in this setting. Evidence on the construct of organizational readiness and tools to measure it in South Africa would help to guide the implementation and ongoing support of interventions intended to improve health system operation.

 In this study, we aimed to assess the performance of a measure of ORIC in a pilot study of health facilities in South Africa. We specifically assessed readiness for the Central Chronic Medicine Dispensing and Distribution (CCMDD) programme. CCMDD was introduced nationally in September 2016, and was intended to improve medication access and reduce facility congestion by enabling individuals with chronic conditions such as HIV or hypertension to pick up longer durations of prescriptions and at more convenient (in terms of time and/or length of wait) pick-up points so long as their health is stable.^8^ Implementation uptake has varied by district,^23^ with promising evidence on effectiveness.^24,25^ In this study, we tested measures of organizational readiness to implement CCMDD in primary care facilities in a rural area of South Africa. We chose the ORIC scale because its brevity makes it suitable for rapid use in clinical settings and because it has been successfully translated and validated in multiple healthcare settings. We hypothesized that better resourced facilities and those where staff place greater value on the intervention would show higher organization readiness to implement CCMDD, and in turn, higher readiness should be associated with uptake of CCMDD and improved service provision for individuals living with chronic conditions such as HIV.

## Methods

###  Study Setting

 This study was conducted in health facilities serving the population covered by the Agincourt Health and Socio-Demographic Surveillance System (Agincourt HDSS) in Bushbuckridge Municipality, Mpumalanga Province, northeastern South Africa. The Agincourt HDSS was established in 1992 to support district health systems development post-apartheid and is run by the Medical Research Council/Wits University Rural Public Health and Health Transitions Research Unit.^26^ The Agincourt HDSS covers approximately 117 000 individuals (20 000 households) in 31 enumerated villages. Prevalence of chronic disease is high: approximately 1 in 5 adults is living with HIV,^27^ over half of adults over 40 have elevated blood pressure, and 10% have diabetes.^28^ As of 2018, 9 public health facilities with a total of 160 nursing positions and 28 lay counselor posts served the villages in the Agincourt HDSS. This includes 6 primary care clinics that provide services 8 hours per day and are each staffed by 8 to 16 nurses and 1 or 2 lay counselors, as well as 3 community health centres that operate 16-24 hours per day and offer additional services such as care for uncomplicated deliveries. The health centres are staffed by 24 to 41 nurses and 4 to 7 lay counselors each. To date, the primary implementation of CCMDD has been the establishment of separate pick-up points within rather than external to each facility for stable patients receiving chronic care.

###  Data Collection

 This pilot study was nested within ongoing research activities in the Agincourt HDSS, including a randomized community mobilization intervention to increase engagement in HIV care and treatment.^29^ We triangulated across three data sources for the purposes of this analysis: an electronic clinical tracking system active from 2015-2018, a comprehensive clinic quality assessment in mid-2018, and provider interviews to pilot test the ORIC measure in early 2019.

 As part of the ongoing research on engagement in HIV care, an electronic clinical tracking system called HDSS-Clinic Link was established in 2015 to capture clinical visits for consenting adults aged 18–49 and link individuals to the population database.^30^ Clinic visit data were supplemented with records from the National Health Laboratory Service on viral load testing. Recognizing that engagement in care could vary within the research site based on the quality of the nearest health facility, a comprehensive quality assessment was conducted at each facility between June and August 2018.^31^ The assessment in each facility included an audit of inputs to care (infrastructure, equipment, medication, supplies) as well as 25 time-motion observations to document wait time and 25 exit interviews with adult patients (18-49 year-olds).

 As an extension of the quality assessment, we conducted a new study from February to May 2019 to pilot the ORIC scales and assess provider ratings of the quality of health services at the facility. Eligible providers included nurses and lay counsellors. The target sample size was 5 per clinic and 8 in each of the health centres to better represent the larger staff at these facilities. We drew a convenience sample based on availability during the 2 to 3 days of data collection in each facility and prioritized nurses as the core clinical staff in this area. Informed consent was obtained from all respondents willing and able to participate. The instrument included items on organizational readiness for CCMDD as well as items on the value of CCMDD to patients.^12,32^ We administered the items in English: prior studies in the area found that providers preferred English over the local language of Shangaan for surveys. Prior to implementation, the field worker reviewed items with members of the MRC/Wits (Agincourt) Public Engagement Office who routinely translate research instruments between English and Shangaan and developed standard explanations in Shangaan to have ready in case providers questioned the meaning of specific words. Organizational readiness was measured after rather than before programme initiation; we make the assumptions that facility staffing is relatively stable and that clinic scores are correlated over time. Our previous study in these facilities found that the average provider had worked at the facility for 5 years,^31^ supporting the assumption that many of the interviewed staff had been in place before the CCMDD programme was introduced.

###  Measures

 We administered the 10-item ORIC instrument as validated by Shea and colleagues.^12^ In line with the instrument design, items from both of its subscales, change commitment and change efficacy, were asked in specific reference to implementation of CCMDD. The change commitment subscale consisted of 5 questions (example item: “We are motivated to implement this program”). Change efficacy was assessed using 5 items (example item: “We can support people as they adjust to this program”). Response options were 5-point Likert scales that ranged from “strongly disagree” to “strongly agree”; providers could also choose “Not applicable.”

 We extracted measures of the factors that may influence ORIC (change valence and resource availability) and result from ORIC (programme uptake, patient wait times, quality of care) from these interviews as well as the clinic quality assessment and Clinic Link data. Details on each measure are summarized in Supplementary file 2 for ease of reference. We asked providers 3 items on the value of the programme to patients (for instance, “This programme has more advantages than disadvantages for patients”) with a 5-point Likert-scale response; we averaged responses across the items and then within clinics to capture one element of change valence. We calculated inputs to care available in each facility following domains outlined by the World Health Organization (WHO) – infrastructure, equipment, medication, and supplies – with a focus on HIV care and treatment.^33^ Using the results from the facility audit in the clinic quality assessment, each facility was scored for input availability based on the average of the proportion of items present in each domain. Scores ranged from 0 (no items present) to 1 (all items present in all domains).

 CCMDD uptake was defined as the percent of eligible patients – non-pregnant individuals on antiretroviral therapy (ART) for HIV with evidence of viral suppression – who had a note or check in their file indicating enrollment in CCMDD between January 1 and September 30, 2018, the most recent months with full HIV visit data in Clinic Link. (The check box for enrollment in CCMDD was added to the data entry form following the programme roll out in 2016). We used the time motion data from the clinic quality assessment to calculate the total number of minutes that patients spent waiting during visits for chronic care. During exit interviews in the clinic quality assessment, patients were asked their agreement on a 4-point scale (strongly disagree, disagree, agree, strongly agree) with the statement, “I waited too long before being seen.” A score of 4 indicates highest satisfaction with wait time for all patients and 1 lowest. We averaged responses by facility to capture patient rating of wait time. We assessed higher quality care based on the percent of providers rating the quality of HIV treatment at their facility as excellent and on two clinical indicators facility performance on HIV care and treatment. The percent of patients testing positive for HIV who linked to care within 30 days (January 1, 2018 – September 30, 2018) and percent of patients starting ART in calendar year 2017 who had a viral load test approximately 6 months after initiation (between 5 and 8 months of initiation, ie, May 1, 2017 – August 1, 2018) was extracted from clinical records. Higher percentages are indicative of higher quality.

###  Analyses

 To assess the performance of the ORIC items, we first screened items based on response rate (selecting a response other than “Not Applicable”) and retained those with at least 80% response to maintain sample size. We conducted exploratory factor analysis (EFA) and used parallel analysis to determine the number of factors from the study sample with higher Eigenvalues than those averaged over 100 draws from randomly generated, uncorrelated variables.^34^ Following previous studies of the ORIC, we assessed items based on a minimum loading of 0.60 and maximum cross-factor loading of 0.30 using the oblique rotation.^12^ We calculated Cronbach alpha for individual-level scale reliability.

 To assess whether the measure was sufficiently reliable at the group (health facility) level to use as an organizational measure, we calculated the r_WG(J)_ index. The r_WG(J)_ index indicates the extent of agreement within group for scales with multiple items; we compared observed variance to expected variance assuming a uniform distribution and a null hypothesis of no clustering by group. An r_WG(J)_ value of 1 indicates perfect agreement. We calculated the intraclass correlation (ICC) of the final measures to express the proportion of variance between groups out of total variance; it ranges from 0 to 1 with higher values indicating greater clustering within relative to between groups.

 For the validity assessment, we calculated facility-level ORIC scores by first averaging provider responses on the final items and then averaging across providers within facility. We compared facility characteristics by facility tier using Kruskal-Wallis tests. We assessed the correlation of ORIC with each factor in our conceptual model at the facility level.

## Results

 All providers approached to participate consented; the target sample of 54 providers was achieved. Fifty-two of 54 were nurses, representing 39% of the 132 nurses employed at these facilities; 4 in 5 were female (Table 1). The median facility score for inputs to HIV care was 0.82 out of 1; gaps in infrastructure and equipment were more common than stock outs of supplies or medication. Most providers somewhat or strongly agreed with statements about the value of CCMDD to patients. Facilities had enrolled 26% of eligible patients into CCMDD during 2018; half of providers rated the quality of HIV treatment at their facility as excellent. Median wait time was 85 minutes for chronic care services; patient ratings on average were closest to the response option of “Agree” that wait time was too long. Nine in ten individuals testing positive for HIV linked with care within 30 days; just over half of those initiating ART had their viral load tested approximately 6 months after initiation per national standards.

**Table 1:** Characteristics of Providers and Health Facilities

**Provider Characteristics (n = 54)**	**No. (%)**
Facility tier	
Clinic	30 (56)
Health centre	24 (44)
Provider cadre	
Professional nurse and operational manager	5 (9)
Professional nurse	32 (59)
Enrolled nurse	15 (28)
Lay counsellor	2 (4)
Provider gender	
Female	45 (83)
Male	9 (17)
**Facility Characteristics (n = 9)**	**Median (Q1, Q3)**
Inputs to care (0 to 1)	0.82 (0.77, 0.89)
Value of CCMDD program to patients (1 worst, 5 best)	4.13 (3.73, 4.53)
CCMDD program uptake	26% (18%, 34%)
Minutes waited for chronic care services	85 (75, 102)
Patient rating of wait time (1 worst, 4 best)	2.36 (2.00, 2.67)
Providers rating HIV treatment as excellent	50% (20%, 80%)
Patients linking to care within 30 days of testing	90% (88%, 94%)
Patients with viral load tested between 5 to 8 months of ART	52% (51%, 56%)

Abbreviations: ART, antiretroviral therapy; CCMDD, Central Chronic Medicine Dispensing and Distribution.

Table 2 shows the performance of the 10-item ORIC instrument. Only 32 of 54 providers provided a valid (non “Not applicable”) response to the item, “We want to implement this program;” this item was dropped from subsequent analysis. Of the 40 providers with valid responses to the remaining 9 items, all strongly agreed with the statement that “We can keep track of progress in implementing this program,” providing no variability for analysis. EFA of the remaining 8 items found 1 Eigenvalue that exceeded 1 (Eigenvalue 3.99); the first Eigenvalue exceeded average Eigenvalues from the parallel analysis by 3.0 (Supplementary file 3). Using a single factor solution, 2 items – “committed to implementing this program” and “can coordinate tasks so that implementation goes smoothly” – failed to load onto the single common factor at 0.60. The remaining 6 items loaded onto the final single-factor model above 0.60; Cronbach alpha for this scale was 0.82.

**Table 2:** Item Performance, ORIC Scales

		**Response Rate**	**Providers Who Strongly Agree**	**Initial Model Factor Loading (n = 40)**	**Final Model Factor Loading (n = 44)**
Change commitment	We are committed to implementing this program	49 (90.7%)	46 (85.2%)	0.34	NA
	We are determined to implement this program	53 (98.1%)	45 (83.3%)	0.92	0.86
	We are motivated to implement this program	53 (98.1%)	48 (88.9%)	0.86	0.86
	We will do whatever it takes to implement this program	49 (90.7%)	40 (74.1%)	0.86	0.73
	We want to implement this program	32 (59.3%)	20 (37.0%)	NA	NA
Change efficacy	We can manage the politics of implementing this program	49 (90.7%)	41 (75.9%)	0.78	0.79
	We can support people as they adjust to this program	53 (98.1%)	49 (90.7%)	0.71	0.66
	We can coordinate tasks so that implementation goes smoothly	54 (100.0%)	52 (96.3%)	0.00	NA
	We can keep track of progress in implementing this program	54 (100.0%)	53 (98.1%)	NA	NA
	We can handle the challenges that might arise in implementing this program	53 (98.1%)	40 (74.1%)	0.67	0.63

Abbreviation: ORIC, organizational readiness to implement change.

 The r_WG(J)_ statistic on average was 0.96, ranging from 0.83 to 1.00 for individual facilities, indicating strong agreement within each facility. With this evidence of interrater reliability within facilities, we aggregated provider scores on the 6-item scale to the facility level. The ICC of this scale was 0.23, indicating that 23% of total variance was between providers at different facilities.

 The aggregated 6-item ORIC scale was high at all facilities, with an average of 4.76 (standard deviation 0.29) out of a maximum possible of 5. Within this narrow range, validation assessment showed that facilities with higher inputs to HIV care had higher ORIC, as did facilities where providers felt that CCMDD was valuable to patients. Higher ORIC was significantly correlated with greater patient satisfaction with wait time and a higher proportion of patients linking to care within 30 days of a positive HIV test (Table 3). Facility-level ORIC did not show a correlation with other hypothesized outcomes, including uptake of CCMDD in the study facilities or observed time waited for chronic care services.

**Table 3:** Correlation of 6-Item ORIC Scale With Facility Factors (N = 9 Facilities)

	**Pearson’s Correlation Coefficient (** ***P*** ** Value)**
Inputs to HIV care	0.62 (.07)
Perceived value of CCMDD to patients	0.68 (.05)
CCMDD uptake	0.07 (.86)
Minutes waited for chronic care services	-0.06 (.88)
Patient rating of wait time (0 worst, 1 best)	0.70 (.04)
Providers rating HIV treatment as excellent	0.38 (.31)
Patients linking to care within 30 days of testing	0.70 (.04)
Patients on ART with viral load tested between 5 and 8 months of initiation	-0.38 (.31)

Abbreviations: ART, antiretroviral therapy; CCMDD, Central Chronic Medicine Dispensing and Distribution; ORIC, Organizational readiness to implement change.

## Discussion

 This pilot study of measures of ORIC in health facilities in Bushbuckridge Municipality, South Africa, found that a subset of items from an existing ORIC measure loaded onto a single factor and showed reliability at the individual level as well as agreement within facility. Support for the validity of this construct based on correlation with other facility factors was mixed. While the study sample is small, it represents nearly 4 in 10 nurses in these health facilities. These findings show promise for the measurement of ORIC in the South African context while pointing to a need for further adaptation and validation.

 Item assessment identified two items that did not perform well in this context: for the first, over 40% of providers responded “Not applicable” when asked about wanting the CCMDD programme in place. This may reflect that CCMDD is established national policy and that providers are not in a position of deciding on programme implementation, or in rare cases that providers were not working at the facility at the time of its introduction. Similarly, all but 1 provider strongly agreed that the facility could keep track of the program; this uniform response likely reflects experience in implementing the programme to date and the emphasis on progress reporting for priority initiatives.

 Of the remaining 8 items, EFA found that 6 loaded strongly onto a single factor; the factor loadings ranged from 0.63 to 0.92. While the magnitude of item loading is not dissimilar from previous studies,^12^ prior studies in Western contexts have supported the original hypothesis of 2 factors – change commitment and change efficacy – for these items.^12,17,18^ The difference in this study may reflect less distinction or a finer nuance between commitment and efficacy in this setting, or diminished difference between these constructs following introduction of the CCMDD program. Further exploration of the domains of the organizational readiness to change theory in this setting and adaptation of the scales is warranted. A particular priority is the addition of items that would be less likely to be endorsed to increase sensitivity at the lower end of the scale.

 Using the items that loaded strongly onto a single factor, we assessed reliability at the individual and facility levels. The 6 items had good reliability for individual providers (alpha 0.82). The r_WG(J)_ values showed sufficient agreement for each facility to aggregate provider responses. These results are comparable to the findings of interrater reliability for each subscale in the original development of the ORIC measure.^12^ The ICC of 0.23 suggests moderate clustering of ORIC scores within facilities, comparable to the ICC found when ORIC scales were used in nursing units in Swiss hospitals.^19^ As a whole, this evidence supports the reliability of the scale within individual respondents and for use at an organizational level.

 With the limited sample of 9 fairly similar facilities, the validation analysis found mixed support for our hypotheses of the determinants and outcomes of ORIC in this setting. Facilities with better inputs to HIV care and where providers saw greater value in CCMDD for patients had higher clinic-level ORIC; higher ORIC was correlated with more positive patient ratings of wait time and with greater linkage to care following a positive HIV test. However, we did not find evidence that the CCMDD programme itself had greater uptake at facilities where providers assessed readiness for this programme to be higher.

 The primary limitation of this work is the timing of the pilot assessment of ORIC measures after the implementation of the CCMDD programme and after the collection of most clinic quality measures. Provider responses were likely affected by experience with CCMDD; if responses differed from pre-implementation ORIC in ways that were systematically related to the quality outcomes, correlations between measures would be biased. We did not ask individual providers if they were working at the same facility when CCMDD was introduced to ensure their responses reflected the experience of their current facility, though our previous research suggests most respondents would have been in the same facility for several years. Measures used for validity assessment may not fully capture concepts such as change valence or availability of resources. Selection of a tool with more domains pertaining to organizational readiness could have yielded a broader view of health facility functioning. Findings are further limited by the small number and relative homogeneity of the study facilities, the lack of qualitative exploration of the constructs and items, and potential incompleteness of routine clinical data.

## Conclusion

 Prior research on organizational readiness measures has been conducted largely in the US and Europe^10,16^; this study is one of the first to provide empirical evidence on the performance of organizational readiness measures in healthcare settings in South Africa and one of a small number of studies in any country to link measures of ORIC to external measures of facility performance.^13,15^ Pilot findings support a unidimensional construct of organizational readiness that was reliable at the individual and facility level and was correlated with perceived value of the program, inputs to HIV care, patient ratings of wait time, and greater uptake of HIV care following testing. While other elements of our hypothesized model such as higher uptake of CCMDD were not supported, initial results indicate that ORIC could be a useful measure. Further testing and adaptation are warranted to provide tools for guiding and evaluating implementation of critical, evidence-based interventions in the public health sector in South Africa.

## Acknowledgements

 This work was supported by Harvard University Center for AIDS Research, an NIH-funded program (P30AI060354). Data presented herein are supported by the US National Institute of Mental Health (R01MH103198). The Agincourt longitudinal research platform is supported by the National Department of Science and Innovation, South African Medical Research Council and University of the Witwatersrand, as well as the Wellcome Trust, United Kingdom (Grants 058893/Z/99/A; 069683/Z/02/Z; 085477/Z/08/Z; and 085477/B/08/Z). The funding institutions have not participated in design or interpretation of findings. The contents are solely the responsibility of the authors and do not necessarily represent the views of the funders. The authors thank Meriam Meritze for her efforts in data collection and the healthcare providers in the participating health facilities for their time and insights.

## Ethical issues

 Approval for this research was provided by the Harvard Human Research Protection Program (IRB18-1400), the Human Research Ethics Committee (Medical) at the University of the Witwatersrand, Johannesburg, South Africa (Ethics Ref No. 150104), and the Provincial Health Research Committee at the Mpumalanga Province Department of Health (MP_201812_004).

## Competing interests

 WTS reports grants from the National Institute of Mental Health (R01MH103198; PI: Lippman) during the conduct of the study. HHL reports grants from Harvard Center for AIDS Research during the conduct of the study and grants from Bill and Melinda Gates Foundation, outside the submitted work.

## Authors’ contributions

 Conception and design: HHL, RW, RT, WTS, and SAL. Acquisition of data: HHL, RW, and NM. Analysis and interpretation of data: HHL, RT, NM, WTS, and SAL. Drafting of the manuscript: HHL. Critical revision of the manuscript: RW, RT, NM, WTS, KK, and SAL. Statistical analysis: HHL. Obtaining funding: KK, SAL, and HHL. Administrative, technical, or material support: RW. Supervision: RT and SAL.

## Supplementary files

Supplementary file 1. Conceptual Framework.

Supplementary file 2. Summary of Measures for Analysis.

Supplementary file 3. Scree Plot and Parallel Analysis Results.
