# Supplementary material for: Measuring Organizational Readiness for Implementing Change in Primary Care Facilities in Rural Bushbuckridge, South Africa
Source: Int J Health Policy Manag. 2020 Nov 23;11(7):912–8. doi: 10.34172/ijhpm.2020.223 (PMC9808169; doi:10.34172/ijhpm.2020.223)
Supplement: Supplementary file 1 — Conceptual Framework. [file ijhpm-11-912-s001.pdf]

Supplementary file 1. Conceptual Framework

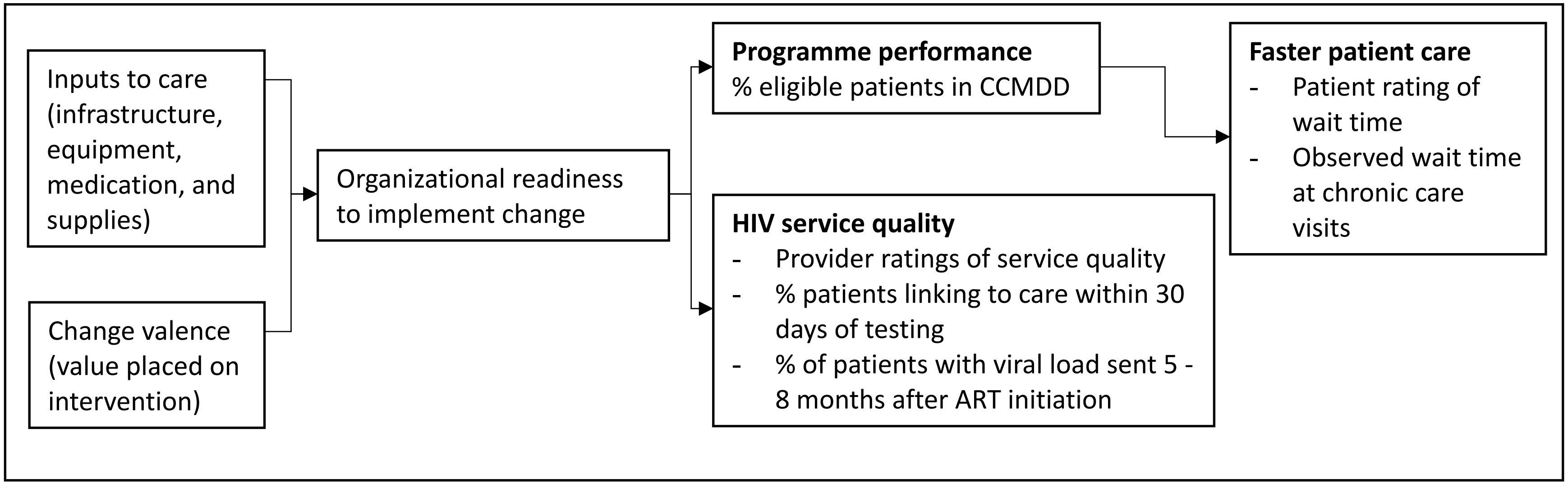

Abbreviations: ART, antiretroviral therapy; CCMDD, Central Chronic Medicine Dispensing and Distribution.
