# Supplementary material for: Measuring Organizational Readiness for Implementing Change in Primary Care Facilities in Rural Bushbuckridge, South Africa
Source: Int J Health Policy Manag. 2020 Nov 23;11(7):912–8. doi: 10.34172/ijhpm.2020.223 (PMC9808169; doi:10.34172/ijhpm.2020.223)
Supplement: Supplementary file 2 — Summary of Measures for Analysis. [file ijhpm-11-912-s002.pdf]

Supplementary file 2. Summary of Measures for Analysis

**Measure**	**Data Source and Dates**	**Calculation**	**Range**
Facility organizational readiness to implement change (ORIC)	Pilot study of 54 providers in 9 facilities, Feb-May 2019	1. Provider score is an average of responses (1 = strongly disagree, 5 = strongly agree) to 6 statements on change commitment and change efficacy<br>2. Facility score is average of responding providers	1 (lowest organizational readiness, all providers strongly disagree with all items) to 5 (highest organizational readiness, all providers strongly agree with all items)
Perceived value of CCMDD to patients	Pilot study of 54 providers in 9 facilities, Feb-May 2019	1. Provider score is an average of responses (1 = strongly disagree, 5 = strongly agree) to 3 statements on value of CCMDD to patients<br>2. Facility score is average of responding providers	1 (lowest value, all providers strongly disagree with all items) to 5 (highest value, all providers strongly agree with all items)
Inputs to care	Facility audit, clinic quality assessment Jun – Aug 2018	1. Proportion of items present calculated per domain for infrastructure, equipment, medication, and supplies<br>2. Average calculated across 4 domains per clinic	0 (no items present in any domain) to 1 (all items present in all domains)
CCMDD uptake	Clinic Link routine data, Jan 1 – Sep 2018	Numerator: non-pregnant individuals receiving antiretroviral therapy at that facility and in stable condition with a note or	0% (no eligible patients enrolled in CCMDD) to 100% (all eligible patients enrolled in CCMDD)

		check indicating enrollment in CCMDD<br>Denominator: all non-pregnant individuals receiving antiretroviral therapy at that facility and in stable condition	
Wait time for chronic care	Time motion observations of chronic care patients, clinic quality assessment Jun – Aug 2018	Median time waited (minutes) for patients receiving any chronic care service within each facility	Not restricted; individual patients waited 4 minutes to 273 minutes (median 80.5)
Patient satisfaction with wait time	Exit interviews with chronic care patients, clinic quality assessment Jun – Aug 2018	1. Patient response to item "I waited too long before being seen" reverse coded so 1 = strongly agree, 4 = strongly disagree<br>2. Responses averaged within facility	1 (lowest rating of wait time, all patients strongly agree wait time too long) to 4 (highest rating of wait time, all patients strongly disagree wait time too long)
Higher quality care: provider rating of HIV treatment quality	Pilot study of 54 providers in 9 facilities, Feb-May 2019	Numerator: providers rating the quality of HIV treatment services at the facility as excellent<br>Denominator: all interviewed providers at the facility	0% (no providers rate care quality as excellent) to 100% (all providers rate care quality as excellent)
Higher quality care: adherence to national guidelines for HIV care – linkage to care	Clinic Link routine data, Jan 1 – Sep 2018	Numerator: patients testing positive for HIV at the facility who initiated HIV care within 30 days<br>Denominator: patients testing positive for HIV at the facility	0% (no patients with positive HIV tests linked to care within 30 days) to 100% (all patients with positive HIV tests linked to care within 30 days)
Higher quality care: adherence to national guidelines for	Clinic Link routine data, Jan 1 2017 – Sep 2018	Numerator: patients starting ART between Jan 1 and Dec 31 2017 at the facility with viral load	0% (no patients initiating ART administered viral load test approximately 6 months after initiation) to

HIV treatment – viral load testing		tested between 5 and 8 months after initiation<br>Denominator: patients starting ART between Jan 1 and Dec 31 2017 at the facility	100% (all patients initiating ART administered viral load test approximately 6 months after initiation)

Abbreviations: ART, antiretroviral therapy; CCMDD, Central Chronic Medicine Dispensing and Distribution.
