# Supplementary figures and images for: Measuring Organizational Readiness for Implementing Change in Primary Care Facilities in Rural Bushbuckridge, South Africa
Source: Int J Health Policy Manag. 2020 Nov 23;11(7):912–8. doi: 10.34172/ijhpm.2020.223 (PMC9808169; doi:10.34172/ijhpm.2020.223)

**Supplementary file 3. Scree Plot and Parallel Analysis Results**

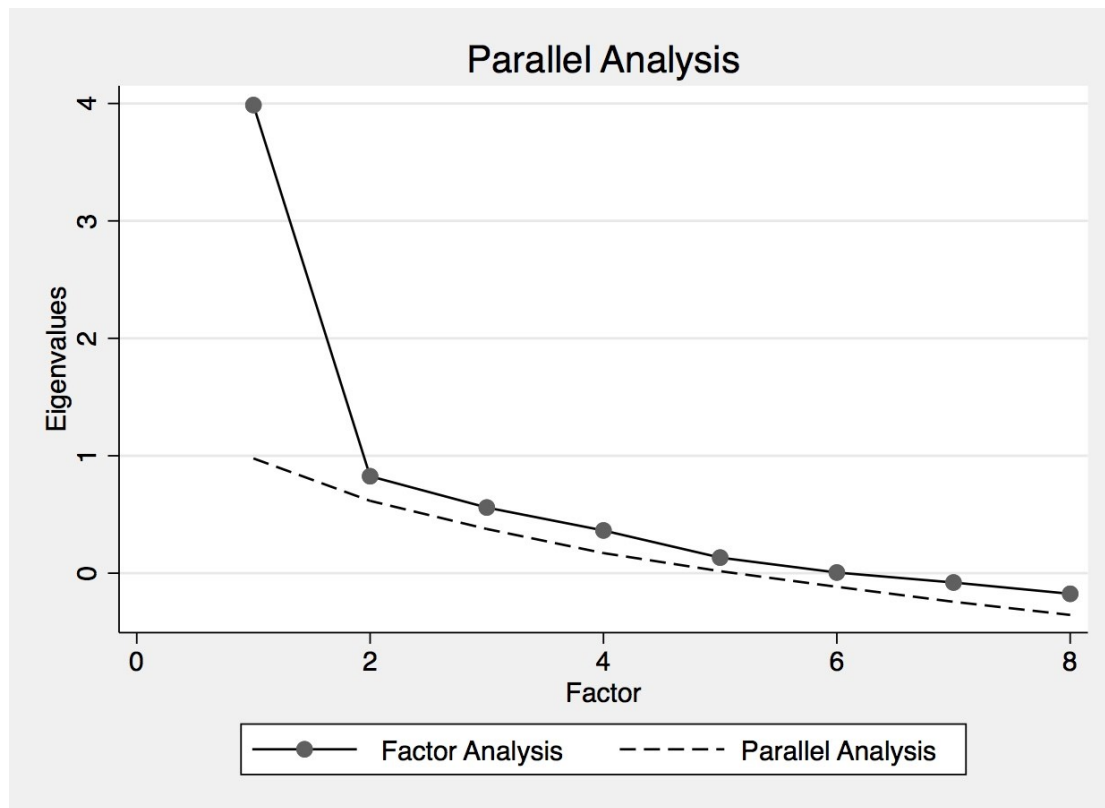

Supplement: Supplementary file 3 — Scree Plot and Parallel Analysis Results. [file ijhpm-11-912-s003.pdf]
